# Evidence from checklists for a Holarctic (circumboreal) kingdom of diatoms

**DOI:** 10.3897/phytokeys.108.26277

**Published:** 2018-08-13

**Authors:** Loren Bahls

**Affiliations:** 1 Montana Diatom Collection, 1032 12th Avenue, Helena, MT 59601 USA Montana Diatom Collection Helena United States of America

**Keywords:** diatoms, North America, Holarctic, biogeography, cosmopolitan, endemic, native

## Abstract

Published checklists of freshwater diatoms that represent the American Northwest, Laurentian Great Lakes, Germany and the South Polar Region were compared systematically and the numbers of taxa shared by two or more of these regions were noted. There is a higher level of floristic correspondence between the American Northwest and Germany (71%) and between the American Northwest and the Laurentian Great Lakes (64%) than between the American Northwest and the South Polar Region (45%). These findings support a Holarctic Kingdom of diatoms that is parallel to the Holarctic Kingdom of flowering plants. Mountains and coastal areas and/or inland waters of high salinity may explain why the American Northwest and Germany have more taxa in common than the American Northwest and the Laurentian Great Lakes. Common riverine diatom taxa in the American Northwest are similar to those reported from nationwide monitoring stations. The number of truly cosmopolitan species – those found on all continents – is probably less than 300. The terms “cosmopolitan”, “endemic” and “native” are often misused when applied to diatoms and the first two terms always need to be qualified.

## Introduction

Until the end of the last century, the prevailing view of freshwater diatom biogeography had been one of cosmopolitan distribution, where the majority of species occur worldwide in suitable habitats and endemism is exceptional. This view assumed that diatoms and other eukaryotic microbes were dispersed globally with few restrictions (Finlay 2002, Finlay et al. 2002). During this era, light microscopy was the primary taxonomic tool and similar taxa were often “lumped” into a single species (e.g., Patrick and Reimer 1966, 1975, Krammer and Lange-Bertalot 1986, 1988, 1991a, 1991b).

This era ended with the widespread availability of electron microscopy, which accelerated the description of new diatom species, most of which appear to be endemic. Several lines of evidence have shown that dispersal of viable diatoms is problematic, that endemism is commonplace, and that geologic history and evolution are just as important as environmental factors in explaining the distribution of diatom species (Edlund and Jahn 2001, Kociolek and Spaulding 2000, Potapova and Charles 2002).

Evolutionary studies of diatom lineages with restricted distributions demonstrate that many regions have unique floristic elements (Kociolek and Spaulding 2000). This suggests that diatom distributions follow the generalized tracks of Croizat (Cox and Moore 2005), which result from tectonic history (vicariance) rather than random dispersal. For example, Ehrenberg (1849, 1850) noted that the flora of western North America was more like that of eastern Asia than the flora of eastern North America. On the Pacific Rim of western North America, the Cascade Mountains are rich in endemic diatom species compared to other regions (Sovereign 1958, 1963), especially in the genera *Gomphoneis* Cleve (Kociolek and Stoermer 1986, 1988) and *Navicula* Bory de Saint-Vincent (Bahls 2011, 2014, Bahls and Potapova 2015, Schmidt 1874–1959).

European floras (e.g., Krammer 2002, 2003) are widely used in the United States and contain many taxa that are common to both Europe and North America. They often refer to “Holarctic”, “northern-alpine”, “circumboreal”, or “Northern Hemisphere” distributions. The monograph by Lange-Bertalot (2001) on *Naviculasensu stricto* is an example of diatom taxa shared by the two continents and the usefulness of European floras in North America: of the 128 taxa addressed by Lange-Bertalot, 110 have been identified from the northwestern United States (Bahls 2009). The occurrence of many diatom species in both North America and Europe suggests a biogeographic model for diatoms that conforms to the Holarctic Kingdom of flowering plants (Takhtajan 1986).

Other taxa that were thought to be endemic to North America or Europe are showing up on both continents. For example, *Amphoracalumetica* (Thomas *ex* Wolle) Peragallo and *Distrionellaincognita* (Reichardt) Williams were assumed to be endemic to the Laurentian Great Lakes and to northern Europe, respectively (Edlund and Stoermer 1999, Reichardt 1988, Krammer and Lange-Bertalot 1991a). However, *A.calumetica* was subsequently found in lakes of northern Europe (Reichardt 2000) and *D.incognita* was reported from the Rocky Mountains of North America (Morales et al. 2005).

Systematic comparisons of large diatom checklists are rarely undertaken because they are tedious, especially when the checklists do not exist in electronic format. In addition, identifications of taxa within checklists may be questionable because they often don’t rely on type material or original descriptions. Comparisons are further complicated when different checklists use different names (synonyms) for the same taxon. Nevertheless, diatom checklists contain many hundreds of morphologically distinct taxa that are easily identified and as such they provide a wealth of useful biogeographic information. Four regional diatom checklists from North America, Europe, and the southern hemisphere are compared here to test the Holarctic Kingdom model of diatom biogeography.

## Methods

Four published diatom checklists were systematically compared in this study, each representing a distinct geographic region (Table 1): American Northwest (ANW, combined Bahls 2009 + Prescott and Dillard 1979), Laurentian Great Lakes (LGL, Stoermer et al. 1999), Germany (GER, Lange-Bertalot and Steindorf 1996) and south Polar regions (SPR, Kellogg and Kellogg 2002). These checklists are roughly contemporary, contain about the same number of taxa and represent land areas of similar size. The three regions in the Northern Hemisphere span approximately the same temperate latitudes.

The combined American Northwest checklist was also compared to a diatom dataset produced for rivers of the contiguous United States by the US Geological Survey’s National Water Quality Assessment (NAWQA) Program (Potapova and Charles 2002), which allowed for a comparison of the most frequently occurring taxa in the two datasets.

Because the combined ANW checklist is newer than the others and contains taxa that were described after the other checklists were published, it had to be adjusted to reflect the taxonomy in place at the time the other checklists were prepared. For comparison with the LGL and GER checklists, diatom names with a publication date of 1997 or later were removed from the ANW checklist (Table 1). For comparison with the SPR checklist, names with a publication date of 2001 or later were removed from the ANW checklist.

**Table 1.** Characteristics of diatom floras compared in this paper (NAWQA=National Water Quality Assessment Program; ANSP=Academy of Natural Sciences of Philadelphia).

**Flora**	**References**	**Latitude range**	**Total names**	**Synonyms**	**Total taxa**	**Unknowns**	**Named taxa^1^**	**Genera^2^**
American Northwest (12/31/1996)	Bahls 2009, Prescott and Dillard 1979	38–49°N	1989	549	1440		1440	
American Northwest (12/31/2000)	Bahls 2009, Prescott and Dillard 1979	38–49°N	2125	622	1503		1503	
American Northwest (08/31/2008)	Bahls 2009, Prescott and Dillard 1979	38–49°N	2552	637	1915	334	1581	120
Laurentian Great Lakes (LGL)	Stoermer et al. 1999	40–50°N	2188	716	1472		1472	124
Germany (GER)	Lange-Bertalot and Steindorf 1996	47–55°N			1632		1632	128
South Polar Region (SPR)	Kellogg and Kellogg 2002	35–90°S			1526		1526	105
NAWQA (ANSP)	Potapova and Charles 2002	25–49°N			1548	381	1167	

^1^At the species and infra-species level.

^2^Non-marine genera.

Synonyms were counted as a match. For example, if one list contained *Achnanthidiumminutissimum* (Kützing) Czarnecki and the other list had *Achnanthesminutissima* Kützing (but not *Achnanthidiumminutissimum*), this was counted as one taxon in common to both lists. For genera, old names were brought up-to-date with combinations used in the ANW list. For example, the GER list contained *Naviculaminima* Grunow and this same taxon is listed as *Eolimnaminima* (Grunow) Lange-Bertalot in the ANW list, hence *Eolimna* was counted as a genus that is common to both lists.

The SPR checklist (Kellogg and Kellogg 2002) contains a number of taxa from marine littoral areas. Strictly marine genera (e.g., *Hyalodiscus*, *Licomorpha*, *Paralia*) and their included taxa were removed from the SPR list prior to comparisons. Marine species in other genera (e.g., *Melosira*, *Tabularia*, *Tropidoneis* [*Plagiotropis*]) that include both marine and freshwater representatives were also removed.

## Results

### Regional checklists compared

At the sub-genus level, the ANW shares from 676 (SPR) to 1017 (GER) taxa with the three other checklists that were compared in this study (Table 2). Notably, the 1017 taxa shared with Germany represent 71% of the taxa reported from the ANW. Curiously, the ANW shares about 100 more taxa with Germany than it does with the much closer Laurentian Great Lakes region (916 taxa shared).

Although the ANW, LGL, and SPR checklists are nearly equal in size (the SPR list is slightly larger), there were many more matches between ANW and LGL (916) than between ANW and SPR (676). Matches between ANW and LGL represent 64% of the ANW flora, whereas matches between ANW and SPR represent only 45% of the ANW flora. This 45% agreement might seem large given that the ANW and South Polar regions are on nearly opposite ends of the Earth, however the SPR checklist extends to 35°south latitude and includes temperate as well as sub-Antarctic and Antarctic habitats (Kellogg and Kellogg 2002). Also, researchers working on samples from the Southern Hemisphere rely mostly on floras prepared for the Northern Hemisphere and may be inclined to “force” southern specimens into northern taxa.

At the genus level there is much more agreement among checklists (Table 2). When the GER and ANW checklists are compared, the 114 genera they have in common represent 95% of the 120 genera reported from the American Northwest. The 109 genera in common between LGL and ANW represent 91% of the 120 genera reported from ANW and the 106 genera shared by SPR and ANW represent 88% of the ANW genera. Even when all four checklists are compared, the 96 shared genera represent 80% of the genera present in the ANW checklist. This suggests a lower rate of endemism at the genus level than at the sub-genus level.

When all three checklists from the Northern Hemisphere are compared, there is a considerable decline in the number of shared taxa to 472 or 33% of the taxa in the ANW checklist (Table 2). These may be core or “signature” taxa that define the Holarctic Kingdom of diatoms. When all four checklists are compared, the number of shared taxa declines to 309, which is only 21% of the sub-generic taxa in the ANW checklist. These taxa are widespread in temperate regions of both the Northern and Southern Hemispheres. If comparable checklists of taxa from temperate regions of Africa, Asia and South America were also included, the list of truly cosmopolitan taxa (found on all continents) would be shorter, perhaps much shorter.

**Table 2.** Comparing the American Northwest flora with other diatom floras in the Northern and Southern Hemispheres. (ANW=American Northwest; LGL=Laurentian Great Lakes; GER=Germany; SPR=South Polar Region).

Floras compared	Number of taxa in common		Shared taxa as a % of ANW taxa	
	Species/varieties/forms	Genera	Species/varieties/forms	Genera
ANW * LGL	916	109	63.6%	90.8%
ANW * GER	1017	114	70.6%	95.0%
ANW * SPR	676	106	45.0%	88.3%
ANW * LGL * GER	472	103	32.8%	85.8%
ANW * LGL * GER * SPR	309	96	20.6%	80.0%

### ANW flora compared to NAWQA

The NAWQA dataset includes 1167 named taxa and 381 unknowns based on 2735 samples collected at sites scattered across the contiguous United States but concentrated in the eastern part of the country (Potapova and Charles 2002). The combined ANW checklist contains 1581 named taxa plus 334 unknowns based on more than 8,500 samples collected across the ANW, but mostly in Montana (Bahls 2009). All of the NAWQA sites are flowing-water sites, whereas the ANW checklist includes taxa from a wide variety of habitats, including springs, seeps, ephemeral pools, rivers, streams, lakes and wetlands.

The most frequently occurring diatom taxa in the ANW and their corresponding frequency and rank in NAWQA samples are presented in Table 3. Most of these taxa are widespread in temperate regions worldwide and all of them are addressed in the *Freshwater Flora of Middle Europe* (Krammer and Lange-Bertalot 1986, 1988, 1991a, 1991b). As would be expected of taxa with wide geographic ranges, the taxa in Table 3 have broad ecological amplitudes. Twenty-four of these same taxa are among the 30 most frequently occurring taxa at NAWQA sampling sites. Differences between the two lists may be expected given the different geographic scopes of the two datasets and additional habitats represented by the ANW list.

Many of the unknown taxa from both the NAWQA and ANW datasets are probably undescribed and new to science. Most of these will likely have limited distributions and be endemic at one scale or another (local, regional, continental or hemispheric).

**Table 3.** The most frequently occurring diatom taxa in the American Northwest (ANW) (Bahls 2009) and corresponding frequency and rank in National Water Quality Assessment (NAWQA) Program samples (Potapova and Charles 2002). NAWQA and ANW ranks are based on percent of samples in which the taxon was encountered.

**Rank**	**Taxon name**	**ANW % samples**	**NAWQA % samples**	**NAWQA rank**
1	*Achnanthidiumminutissimum* (Kützing) Czarnecki	88.1	59.7	1
2	*Cocconeisplacentula* Ehrenberg	79.7	37.8, 26.8^1^	7, 16^1^
3	*Nitzschiadissipata* (Kützing) Grunow	65.2	22.6	21
4	*Naviculacryptotenella* Lange-Bertalot	64.1	39.8	5
5	*Synedraulna* (Nitzsch) Ehrenberg	60.9	16.7	30
6	*Achnantheslanceolata* (Brébisson) Grunow	58.7	26.4	17
7	*Fragilariavaucheriae* (Kützing) Petersen	56.1	20.8	25
8	*Nitzschiapalea* (Kützing) W. Smith	53.7	32.2, 17.0^2^	10, 28^2^
9	*Gomphonemaparvulum* (Kützing) Kützing	47.4	41.1	3
10	*Amphorapediculus* (Kützing) Grunow	47.3	39.4	6
11	*Encyonemasilesiacum* (Bleisch *ex* Rabenhorst) Mann	44.4		
12	*Naviculaminima* Grunow	42.8	41.0	4
13	*Nitzschiainconspicua* Grunow	42.5	36.3	8
14	*Reimeriasinuata* (Gregory) Kociolek & Stoermer	42.1	31.0	12
15	*Nitzschialinearis* (Agardh) W. Smith	42.0		
16	*Naviculatripunctata* (O.F. Müller) Bory	39.7	22.5	22
17	*Gomphonemapumilum* (Grunow) Reichardt & Lange-Bertalot	39.4	29.1	14
18	*Gomphonemaminutum* (Agardh) Agardh	38.6		
19	*Fragilariacapucina* Desmazières	38.1		
20	*Naviculareichardtiana* Lange-Bertalot	37.9		
21	*Naviculacapitatoradiata* Germain	36.5	27.5	15
22	*Nitzschiafonticola* Grunow	35.8		
24	*Staurosiraconstruens* (Ehrenberg) Williams & Round	35.5		
25	*Cymbellaexcisa* Kützing (=*C.affinis* sensu Patrick & Reimer)	35.0	16.8	29
26	*Rhoicospheniaabbreviata* (Agardh) Lange-Bertalot	34.7	42.6	2
27	*Cyclotellameneghiniana* Kützing	34.6		
28	*Cocconeispediculus* Ehrenberg	34.1	18.1	27
29	*Naviculagregaria* Donkin	33.5	29.4	15
30	*Nitzschiapaleacea* (Grunow) Grunow	33.5		
31	*Gomphonemaolivaceum* (Hornemann) Brébisson	31.7		
32	*Diatomamesodon* (Ehrenberg) Kützing	29.0		
33	*Epithemiasorex* Kützing	28.9		
34	*Caloneisbacillum* (Grunow) Cleve	28.2		
35	*Melosiravarians* Agardh	27.3	24.3	18
36	*Meridioncirculare* (Greville) Agardh	26.7		
37	*Naviculaveneta* Kützing	25.7		
38	*Hannaeaarcus* (Ehrenberg) Patrick	24.4		
39	*Epithemiaturgida* (Ehrenberg) Kützing	24.4		
40	*Rhopalodiagibba* (Ehrenberg) O. Müller	23.8		
41	*Nitzschiaamphibia* Grunow	23.7	33.2	9

^1^*Cocconeisplacentulavar.euglypta* (37.8%) and *C.placentulavar.lineata* (26.8%) were counted separately in NAWQA samples.

^2^*Nitzschiapaleavar.palea* (32.2%) and *Nitzschiapaleavar.debilis* (17.0%) were counted separately in NAWQA samples.

## Discussion

### Evidence for a Holarctic Kingdom of diatoms

Two lines of evidence presented here support a Holarctic distribution model for freshwater diatoms: (1) a high level of floristic affinity between the ANW and Germany, with shared sub-generic taxa accounting for 71% of the ANW flora; and (2) a much lower level of floristic affinity between the ANW and the South Polar Region (45% of sub-generic taxa) than between the ANW and the Laurentian Great Lakes (64%) or between the ANW and Germany. The percentages of genera shared by the regions are larger, ranging from a high of 95% (ANW vs. GER) to a low of 88% (ANW vs. SPR).

There are three possible explanations why the ANW shares more taxa with Germany than it does with the Great Lakes region: (1) The German checklist includes 160 more taxa than the LGL checklist and therefore provides more opportunities for matches; (2) Germany and the ANW both have mountains higher than 2,000 m elevation, which allow for more southerly range extensions of northern-alpine taxa (the Laurentian Great Lakes region lacks high mountains); and (3) Germany and the ANW both have marine coasts and many brackish to hyper-saline inland waters, features that are either not present or not as common in the Great Lakes region. Many salt-tolerant taxa that are common to both Germany and the ANW are addressed in a monograph on diatoms of marine coasts (Witkowski et al. 2000).

Potapova and Charles (2002) note that 1016 (70%) of the 1461 non-planktonic diatom taxa in the NAWQA dataset have been recorded on continents other than North America, mainly Europe. In addition, 49 taxa known only from North America and 15 taxa known only from the New World are included in the NAWQA dataset (Potapova and Charles 2002). A similar but undetermined number of North American and New World endemics also occur in the ANW checklist, including many of those described by Sovereign (1958, 1963) and Kociolek and Stoermer (1986, 1988).

Reinforcing the relative dissimilarity of floras from the Northern and Southern Hemispheres are the findings of Kociolek and Spaulding (2000). They compared lists of diatoms from the Arctic and Subarctic Region of North America to lists from the Antarctic that were presented at a workshop on Arctic-Antarctic diatoms (e.g., Håkansson and Jones 1994, Hamilton et al. 1994). The Arctic and Antarctic regions have aquatic habitats with similar physical conditions at comparable latitudes and may be expected to have similar diatom floras under a cosmopolitan model for diatom distributions. However, only 80 (8.9%) of 897 taxa reported from the two regions are common to both regions (Kociolek and Spaulding 2000).

A Holarctic model for the distribution of diatoms is further supported by established Holarctic distributions for flowering plants (Takhtajan 1986). Historically, dispersal of flowering plants, and probably diatoms, was aided by land bridges that connected Europe with North America (through Greenland) until the late Eocene, and Asia with North America (Bering Land Bridge) off and on until the end of the Pleistocene (Cox and Moore 2005). Finally, all areas in the Holarctic region share a common history of glaciation. Since the end of the Pliocene, several advancing continental glaciers essentially stripped the Holarctic of its preexisting flora and resulted in a “drastic modernization” of the flora across the region (Cox and Moore 2005, Pielou 1991). The Holarctic region thereby became common ground for the establishment of a modern, cold-tolerant flora.

Rappaport’s Rule holds that high-latitude organisms have broader geographic ranges than those from the low latitudes (Cox and Moore 2005). The occurrence of broad-range species at higher latitudes could be a consequence of (1) a common tectonic history (Laurasia giving rise to Laurentia and Eurasia); (2) a common history of glaciation in the Northern Hemisphere, leaving only the most adaptable species behind; or (3) greater seasonal fluctuations at higher latitudes, which select for species with broad ecological amplitudes (Cox and Moore 2005), or a combination of these. Whatever the underlying cause or causes, the broad ranges of many diatom taxa in the northern Hemisphere conform to Rappaport’s Rule.

### Cosmopolitan, endemic, and native diatom species

The term “cosmopolitan” is often used offhandedly to describe freshwater diatom taxa that are widely distributed. Webster’s New World Dictionary defines cosmopolitan as “belonging to the whole world, not national or local; at home in all countries or places”. The term therefore implies global distribution, that is, the taxon is found on all continents. But the term is often used without evidence that the taxon is present on all continents.

Lange-Bertalot, as reported by Kociolek and Spaulding (2000), estimates that there are 150–200 taxa that “occur across great ecological and geographic space that has been influenced by urbanization”. It is true that taxa with broad ecological amplitudes tend to have widespread distributions, but we often don’t know if these taxa occur on all continents. Unless we do know this, diatomists should refrain from using the term cosmopolitan and use more precise terminology, e.g., “widespread in the Northern Hemisphere” or “widespread in temperate regions”, assuming the writer has the distribution records to support these statements.

Similarly, for clarity, the term endemic should not be used without qualification. Appropriate modifiers for endemic might include local, regional, and continental, e.g., North American endemic. For example, the diatom species *Cymbellajanischii* (A. Schmidt) De Toni is endemic to the American Northwest region (Bahls 2007). Endemics can occur at all taxonomic levels, but diatom endemics at the genus level or higher are uncommon. An example is the diatom genus *Gomphocymbella* O. Müller, which is endemic to Africa (Kociolek and Spaulding 2000).

There is also some confusion with and misuse of the term “native” as it applies to diatoms. For example, Potapova and Charles (2004) define native diatom species as those “generally confined to the Americas; known only from America, or common in America but found extremely rarely elsewhere”. What they describe here are actually species that are endemic to the Americas, as well as being native to the Americas. Native means occurring before European settlement or before intercontinental travel and commerce became common and widespread. Documented cases of freshwater diatoms introduced from other continents since European settlement are not common (e.g., Edlund et al. 2000, Spaulding et al. 2010, Reid et al. 2012, Kilroy and Bothwell 2011). If one accepts evolution and vicariance or “historical biogeography” as the prevailing mechanisms that explain diatom distributions (as opposed to unrestricted dispersal and “ecological biogeography”), then one must assume that all taxa are native, unless there is evidence to the contrary of their recent introduction.

## Conclusions

1. Freshwater diatoms in the Northern Hemisphere display a Holarctic distribution that conforms to the Holarctic Kingdom of flowering plants; temperate and polar floras in the Northern Hemisphere are distinct from those of the Southern Hemisphere at the sub-generic level.

2. European diatom floras and monographs are useful for identifying at least 70% of the named sub-generic diatom taxa in the northwestern United States.

3. Shared geographic features (mountains, coastal areas, inland brackish waters) may explain why some regions at temperate latitudes in the Northern Hemisphere have higher levels of floristic similarity (ANW and GER) than regions that are much closer together (ANW and LGL).

4. The terms “cosmopolitan”, “endemic”, and “native” are often misused when applied to diatoms; the first two terms always need to be qualified.

5. The number of truly cosmopolitan diatom taxa (native to all continents) is probably less than 300.
